# Clinical and electroretinographic profile of 27 patients with Stargardt disease treated at a hospital in Brazil

**DOI:** 10.5935/0004-2749.20210053

**Published:** 2021

**Authors:** Letícia Schemberger Schafranski, Izabelle Yumi Honda Müller, Mario Teruo Sato

**Affiliations:** 1 Universidade Federal do Paraná, Curitiba, PR, Brazil; 2 Sector of Neuro-Ophthalmology and Ocular Electrophysiology of the Complexo Hospital de Clínicas, Universidade Federal do Paraná, Curitiba, PR, Brazil

**Keywords:** Electroretinography, Retinal diseases, Retinal pigment epithelium, Macular degeneration, Lipofuscin, Eletrorretinografia, Doenças retinianas, Epitélio pigmentado da retina, Degeneração macular, Lipofuscina

## Abstract

**Purpose:**

Stargardt disease is the most common type of juvenile-onset macular dystrophy. It is bilateral and symmetrical in appearance, affects the macula, and its main characteristic is the loss of central vision that starts in the first or second decade of life. The purpose of this study was to describe the profile of the patients evaluated at the Complexo Hospital de Clínicas da Universidade Federal do Paraná, as well as describe the electroretinographic findings with the full-field electroretinogram in these patients.

**Methods:**

An observational, retrospective study was performed by analysis of records and electroretinographic examinations of 27 patients with Stargardt disease and fundus flavimaculatus who were treated at the Complexo Hospital de Clínicas da Universidade Federal do Paraná’s Department of Ocular Electrophysiology and Neuro-Ophthalmology between 1997 and 2014. The patients included in this study presented clinical features, fundus examination and/or electroretinographic findings compatible with Stargardt disease.

**Results:**

The visual acuity in the best eye varied from 0 to 1.6 logMAR (20/20 to 20/800) with an average of 0.89 ± 0.42 logMAR. The age at onset of symptoms varied from since birth to 36 years old (average 19.2 ± 9.2) with the majority of patients having symptom onset in the first or second decade of life. The mean time from the disease’s first symptoms until the diagnosis was 7.3 years. In the fundus examination, every patient presented some kind of abnormality. In the electroretinogram analysis, the majority of patients had results that differed from those of sample controls, i.e., reduced amplitude and increased implicit time in the photopic and scotopic phases.

**Conclusions:**

The visual acuity and the age at symptoms onset in this study were compatible with the natural history of this dystrophy. The typical fundus appearance of Stargardt disease and altered electroretinogram were more frequent because of the delay until diagnosis. New prospective studies are necessary to evaluate these patients based on emergent technologies.

## Abstract (pt)

**Objetivo:**

A doença de Stargardt é a forma mais comum de distrofia macular de início juvenil. É bilateral e simétrica em aparência, afeta a mácula e sua característica principal é a diminuição da visão central que geralmente inicia-se na primeira ou segunda década de vida. O objetivo do estudo é descrever o perfil clínico dos pacientes avaliados no Complexo Hospital de Clínicas da Universidade Federal do Paraná, bem como descrever os achados eletrorretinográficos destes pacientes com o eletrorretinograma de campo total.

**Métodos:**

Foi realizado um estudo observacional retrospectivo, baseado na análise de prontuários e eletrorretinograma de 27 pacientes com Doença de Stargardt e Fundus Flavimaculatus, atendidos em consulta oftalmológica no ambulatório de Eletrofisiologia Ocular e Neuro-Oftalmologia do Complexo Hospital de Clínicas da Universidade Federal do Paraná, entre 1997 e 2014. Os pacientes incluídos no estudo apresentavam quadro clínico, fundoscopia e/ou achados eletrorretinográficos compatíveis com a doença.

**Resultados:**

A acuidade visual no melhor olho variou de 0 a 1,6 logMAR (20/20 a 20/800), com média de 0,89 ± 0,42 logMAR. A idade de aparecimento dos sintomas variou desde o nascimento a 36 anos (19,2 ± 9,2), sendo a maioria nas 1ª e 2ª década de vida. Em relação ao tempo entre o início dos sintomas e o diagnóstico, a média foi de 7,3 anos. Na fundoscopia, todos os pacientes apresentaram alguma alteração. Na análise do eletrorretinograma, a maioria dos pacientes demonstrou resultados que diferem da amostra de pacientes controles, ou seja, amplitudes reduzidas e tempos de culminação aumentados nas fases fotópicas e escotópicas.

**Conclusões:**

A acuidade visual e idade de início de aparecimento dos sintomas encontrados neste estudo são compatíveis com a evolução desta distrofia. Achados fundoscópicos típicos da doença de Stargardt e eletrorretinograma alterados foram mais frequentes em decorrência do atraso no diagnóstico. Novos estudos prospectivos são necessários para avaliar estes pacientes, fundamentando-se em novas tecnologias.

## INTRODUCTION

Stargardt disease is the most common autosomal recessive type of juvenile-onset macular dystrophy, with an estimated prevalence of 1: 8,000 to 1: 10,000 individuals^(1)^. Described in 1909 by Karl Stargardt^(2)^, the disease is bilateral and symmetrical in appearance and its main feature is the decrease in central vision that starts around the first or second decade of life^(3,4)^. The typical finding at the eye fundus is a pigmentary maculopathy, which manifests itself as a decreased foveal reflex, pigment mottling, beaten-bronze reflex, and bull’s eye pigment appearance; furthermore, it can progress to macular atrophy. White-yellowish spots (flecks) also may be present in the fundoscopy exam^(3,5)^.

In 1963, Franceschetti^(6)^ introduced the term fundus flavimaculatus to describe the findings of irregular white-yellowish spots, rounded or pisciform in the posterior pole or extending from the posterior region to the periphery, associated or not with macular alterations. Afterwards, many authors concluded that both Stargardt disease and fundus flavimaculatus are different manifestations of the same disease^(3,7-9)^, which was confirmed as both presentations are caused by mutations in the *ABCA4* gene^(10-11)^.

The *ABCA4* gene encodes a transmembrane protein located in the outer-segment of photoreceptors that is responsible for clearing a retinoid intermediate of the visual cycle. The reduced activity of this gene leads to accu mulation of the toxic component N-retinylideneN-re tinylethanolamine (A2E) in the outer-segment disc membranes and retinal pigment epithelium (RPE) and precipitates cell death and vision loss^(12,13)^. Dominant autosomal forms of the disease also have been described, but are less common^(14,15)^.

Central scotomas are present since the early stages of the disease, while the peripheral fields remain only slightly affected until the involvement is extensive^(12)^. The visual acuity (VA) decreases during the course of the disease, reaching values close to 20/200 or worse. However, patients who present a later onset of the disease may have a better visual prognosis^(16)^.

The full-field electroretinogram (ERG) and the electrooculogram (EOG) may yield normal results when the disease affects only the macula. The progression of al terations in the fundoscopy exam with diffuse, centroperipheral involvement is accompanied by subnormal amplitudes of cones or cones and rods responses and changes in the test of dark adaptation (DA)^(17,18)^. Fluorescein angiography and fundus autofluorescence have aided in the disease’s diagnosis; for example, choroidal silence in fluorescein angiography occurs in 85.9% of cases, although the absence of this signal does not exclude the disease^(19,20)^.

This study sought to describe in detail the clinical features of a relatively rare visual condition. Therefore, the results may provide additional information to help clinicians diagnose Stargardt disease/fundus flavimaculatus based on the anatomical and other functional changes described in the present report, and would be particularly useful if electrodiagnosis is not available.

## METHODS

This quantitative, descriptive, and retrospective study analyzed the medical records and ERG results of patients with suspected Stargardt disease and fundus flavimaculatus who were assessed at an ophthalmological clinical visit at the Department of Ocular Electrophysiology and Neuro-Ophthalmology of the *Complexo Hospital de Clínicas da Universidade Federal do Paraná* (CHC-UFPR). The individuals were assessed from August 1997 to May 2014. The patients included in the study had a clinical picture, fundoscopy, and/or electroretinographic findings compatible with the disease as described prior. Patients were excluded if they had acute vision loss, macular lesion due to toxoplasmosis or use of chloroquine, high myopia (≤-5.00 D), diagnosis of age-related macular degeneration, or diabetic retinopathy with macular involvement. Patients whose files were not found or whose files did not contain sufficient information for diagnostic confirmation were also excluded.

Data regarding anamnesis (sex, age at onset of symptoms, and time elapsed between onset of symptoms and diagnosis), ophthalmologic examination (VA and fundoscopy with a direct ophthalmoscope), and ERG were obtained. VA was classified according to the standardization described by the International Statistical Classification of Diseases and Related Health Problems 10th Revision^(21)^ and was transformed into logarithm of the minimum angle of resolution (logMAR) for calculation of mean and standard deviation. The ERG was performed with the ocular electrophysiology apparatus (EPIC-2000, LKC Technologies, Inc., Gaithersburg, MD, USA) that includes a Ganzfeld dome. The ERG-jet contact lens was used in this examination. Tropicamide and phenylephrine were used and the patients were dark-adapted for 20 to 30 minutes before the ERG.

The ERG recording followed the recommendations of the International Society for Clinical Electrophysiology of Vision (ISCEV) protocol (1999 update)^(22)^. In all steps of the test, the low-cut filters of the amplifier were set at 0.3 Hz and the high-cut filters at 500 Hz, except for the oscillatory potentials when the low-cut filter was set at 75 Hz. For the first step of the protocol, the dome filter was fixed at 24 dB or 2.4 log, all other steps were set at 0 dB. The calibration of the light source was 1.586 cd.s/m^2^.

The registries were obtained as follows: 1) Scotopic White 24 dB Single Flash, a rod-driven response (Scotopic b) triggered by weak stimuli of 2.4 log below the scotopic-calibrated standard flash with a 2-second interval; 2) Scotopic White 0 dB Single Flash, maximum response of dark-adapted eyes to strong 0 dB stimuli with a 10-second interval (aand b-waves); 3) Scotopic White 0 dB Single Flash, oscillatory potentials, with a 15 second interval between stimuli and the low-cut filter set at 75 Hz; 4) Photopic White 0 dB Single Flash, a cone-driven response (Photopic b), with background light of 17-34 cd/m^2^ causing rod suppression and a minimum interval of at least 0.5 seconds between stimuli. In order to register maximum cone responses, patients were not light-adapted; 5) Photopic White 0 dB 30 Hz Flicker, a cone-pathway-driven response to the repetitive light stimulus, with the same background light as the previous step.

The a-wave is the first negative deflection of the ERG and reflects mainly the activity of the photoreceptors. Under dark-adapted conditions, it is primarily rod-driven (scotopic) and, with a rod-saturating background light, it is primarily cone-driven (photopic). The a-wave is followed by a positive b-wave that derives from ON bipolar cells. The amplitude of the a-wave was measured from the baseline to the a-wave trough, and the b-wave amplitude was measured from the a-wave trough to the b-wave peak; both were quantified in microvolts (µV). Implicit times were calculated from the time of the flash until the peak of the waves and were expressed in milliseconds (ms).

The data were analyzed according to control group values from healthy volunteers for a 95% confidence interval obtained from the normatization of the CHC-UFPR data, which are reproduced in table 1^(23)^. The observed values were related to the right eye, which was randomly chosen because the clinical manifestation of the disease is symmetrical in both eyes.

**Table 1:** Control group values (95% confidence interval) for all ERG steps by age group. Amplitude (_µ_v) and implicit time (ms)^a^

			Lower confidence interval				Upper confidence interval		
**ERG Steps**		**LCI < 20**	**LCI < 40**	**LCI <60**	**LCI >60**	**UCI <20**	**UCI <40**	**UCI <60**	**UCI >60**
S24	A	286.73	229.09	238.91	186.39	399.07	326.91	324.89	232.21
S24	IT	90.75	89.59	90.27	93.52	98.85	97.71	105.13	98.68
S0A	A	246.54	299.34	243.56	225.47	338.66	376.26	359.04	309.73
S0A	IT	15.06	16.01	16.27	16.35	18.04	17.89	19.53	18.85
S0B	A	273.83	181.48	242.97	162.81	402.17	329.52	300.83	250.99
S0B	IT	45.88	45.40	48.29	44.59	50.72	49.70	52.91	51.01
OP	A	222.89	227.88	240.14	179.30	322.97	364.50	324.22	303.84
P0	A	57.86	54.01	54.75	50.71	83.34	103.79	85.05	81.69
P0	IT	28.67	29.37	30.32	27.42	30.,93	31.13	33.18	30.28
FL	A	53.21	67.73	68.29	45.79	104.59	105.99	98.09	94.73
FL	IT	24.32	27.55	28.60	19.89	28.98	28.79	30.40	31.13
N		10	10	10	10				
Total					n=40				

LCI= Lower confidence interval; UCI= Upper confidence interval; S24= Scotopic White 24 dB Single Flash; S0A= Scotopic White 0 dB Single Flash (a-wave); S0B= Scotopic White 0 dB Single Flash (b-wave); OP= Scotopic White 0 dB Single Flash-Oscillatory Potentials; P0= Photopic White 0 dB Single Flash; FL= Photopic White 0 dB 30 Hz Flicker; A= Amplitude (µv); IT= Implicit time (ms); <20=Patients aged less than 20 years old; <40= Patients aged 20 to 39 years old; <60= Patients aged 40 to 59 years old; >60-Patients aged 60 years or older.

a = Data collected from: Sato MT= Takahashi WY= Moreira Júnior CA^^23^^.

The research project was approved by the Research Ethics Committee under the number CAAE 24561313.3.0000.0096. The research was done confidentially, and the data collected were used for only academic and scientific purposes.

All the data found were placed in a Microsoft Excel^®^ 12.0 table and the tables and graphs presented in this study were generated using the same software.

## RESULTS

Among the 44 patients initially selected, seven were excluded because of diagnostic doubt and 10 because their medical records were not found by the authors. The remaining 27 individuals corresponded to patients with clinical and electroretinographic features that met the inclusion criteria. Out of the 27 cases selected, 14 (51.9%) patients were female and the mean age was 26.52 ± 12.55 years. Regarding comorbidities, there was one case each of diabetes without retinopathy, a history of epilepsy, congenital nystagmus, peripheral chorioretinal scars, and suspected acute disseminated encephalomyelitis. To avoid confounding bias, these patients were not included in the analysis of the ERG results. One patient had high hypermetropia (≥+5,01 D) and another patient developed strabismus at the age of 15.

Six patients (22.2%) presented fundoscopic alterations compatible with fundus flavimaculatus as an additional clinical feature; however, they were not classified in a separate group.

VA in the best eye ranged from 0 to 1.6 logMAR (20/20 to 20/800). The mean value was 0.89 ± 0.42 logMAR (20/160). According to the classification shown in table 2, eight patients (29.6%) had mild or no visual impairment, nine (33.3%) had moderate visual impairment, seven (25.9%) had severe visual impairment, and two (7.4%) had grade 3 blindness. There were no patients with VA rated as grade 4 or 5 blindness. VA was not determined in only one patient (3.7%).

**Table 2:** Visual acuity classification and distribution of the patients

Visual acuity	Number of patients (%)
0 - Mild or no visual impairment-up to 20/70	8 (29.6)
1 - Moderate visual impairment-20/70 to 20/200	9 (33.3)
2 - Severe visual impairment-20/200 to 20/400	7 (25.9)
3 - Blindness-20/400 to 20/1200	2 (7.4)
4 - Blindness-20/1200 to light perception	0 (0)
5 - Blindness-no light perception	0 (0)
9 - Undetermined or unspecified	1 (3.7)

**Figure 1:**
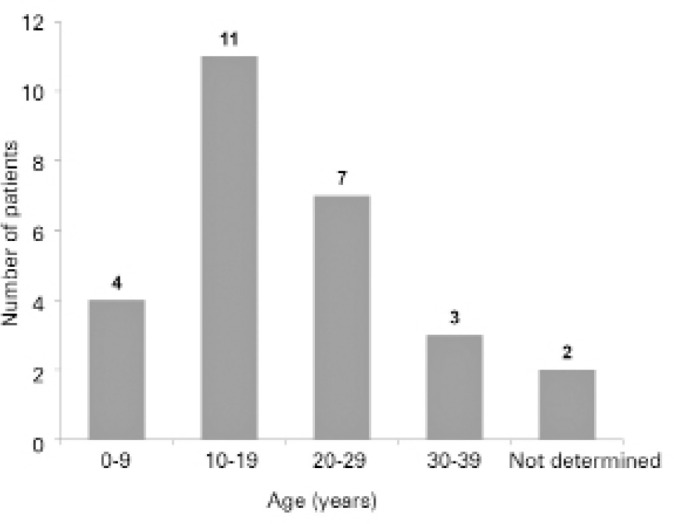
Age at onset of symptoms of Stargardt disease.

The age at symptom onset ranged from birth to 36 years (mean 19.2 ± 9.2 years). Figure 1 shows the distribution of the patients in each age group at symptom onset.

**Figure 2:**
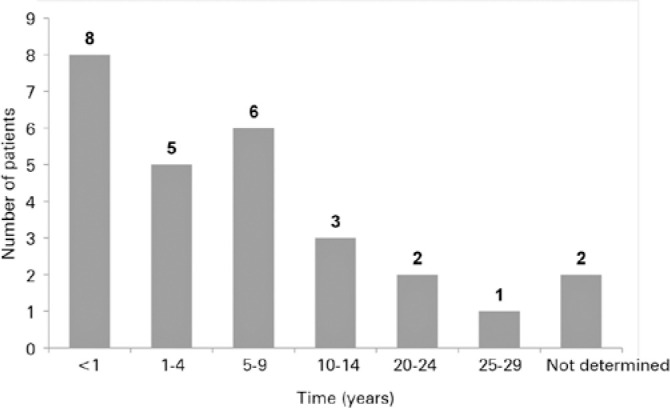
Time between symptoms onset and diagnosis of Stargardt disease.

The mean time between symptom onset and diagnosis was 7.3 years. Figure 2 shows the distribution of the patients according to the time between symptom onset and diagnosis.

In the fundoscopy exam, all patients demonstrated one or more alterations. Eleven patients showed alteration or atrophy in the RPE and six presented a beaten-bronze reflex. Posterior pole spots associated with macular alteration were present in six patients, pigment mottling in four, and absence of foveal reflex in three.

The analysis of the electroretinographic findings is shown in table 3. Except for Step 3 of the exam, in which there is no determination of implicit time, this feature was increased in the majority of patients in all ERG phases. Similarly, the amplitude predominated in a reduced form in the scotopic and photopic phases in most patients.

**Table 3:** Electroretinographic parameters

Electroretinography	Amplitude	Implicit time
**Step 1 -Scotopic White 24 dB Singl** Normal	**e Flash** **3 (13.6%)**	**3 (13.6%)**
Increased	**3(13.6%)**	**12 (54.5%)**
Decreased	**16 (72.7%)**	**7(31.8%)**
**Step 2-Scotopic White 0 dB Single** Normal	**Flash (a-wave)** **3(13.6%)**	**8 (36.4%)**
Increased	**0 (0%)**	**12 (54.5%)**
Decreased	**19 (86.4%)**	**2 (9.1%)**
**Step 2-Scotopic White 0 dB Single** Normal	**Flash (b-wave)** **5 (22.7%)**	**4 (18.2%)**
Increased	**2 (9.1%)**	**18 (81.8%)**
Decreased	**15 (68.2%)**	**0 (0%)**
Step 3^*^-Scotopic White 0 dB Singl	**e Flash-Oscillatory Potentials**	
Normal	**8 (36.4%)**	-
Increased	**0 (0%)**	-
Decreased	**11 (50%)**	-
Undetermined	**3(13.6%)**	-
Step 4^**^-Photopic White 0 dB Single Flash Normal 3(13.6%)		**8 (36.4%)**
Increased	**2 (9.1%)**	**12 (54.5%)**
Decreased	**17(77.3%)**	**2 (9.1%)**
Step 5^**^-Photopic White 0 dB 30 Hz Flicker Normal 8 (36.4%)		**2 (9.1%)**
Increased	**0 (0%)**	**13 (59.1%)**
Decreased	**14(63.6%)**	**7(31.8%)**

* Stage 3 comprises only amplitude.

** Data of time 1’.

## DISCUSSION

The clinical presentations, fundoscopic findings, and progression of Stargardt disease are quite variable. The age at symptoms onset in the patients in this study has corroborated the description proposed by Stargardt because most patients (59%) had their symptoms begin in the first or second decade of life. The mean VA of 0.89 (± 0.42) logMAR was also similar to what is described in the literature^(24,25)^. There was a significant variability in this aspect, from 0 to 1.6 logMAR, that is, mild or no visual impairment (20/20), up to grade 3 blindness (20/800). This variation is explained by the different disease stages of the studied patients, because this disease has a gradual progress^(24)^.

A classification proposed by Fishman^(26)^ in 1976 describes the evolution of findings in fundoscopy and electrophysiologic and psychophysical tests. According to this classification, at the initial stage, there are alterations restricted to the macula, from a mottled appearance to the beaten-bronze reflex, eventually evolving until the atrophy of the RPE and choriocapillaris in the macular region. In most cases, a ring of flecks surrounds a central lesion of atrophic appearance. VA varies between 20/50 and 20/100 and relative or eventually absolute central scotomas may be present. Electrophysiologic tests are often normal at this stage. The second stage is characterized by the appearance of diffuse white-yellowish spots (flecks), similar to those of fundus flavimaculatus, which may undergo partial resorption. Peripheral visual fields, ERG amplitudes, and EOG ratios usually remain normal, although some patients may present a prolonged period for DA.

Stage 3 is characterized by greater reabsorption of the flecks and choriocapillaris atrophy within the macula. Subnormal EOG ratios are present in the majority of cases, and the ERG may demonstrate normal or subnormal cone or cone and rod responses. Diminished DA is also observed. The central field defects are similar to those in previous stages, and the peripheral fields begin to be affected. Stage 4 demonstrates further reabsorption of the flecks and extensive RPE and choriocapillaris atrophy. The peripheral fields are either moderately or severely constricted, and the ERG results exhibit markedly reduced cone and rod amplitudes with alteration on DA testing.

Because of the retrospective nature of this study, it was not possible to classify the patients according to these evolutionary stages; however, all patients showed some kind of fundoscopic alteration, probably related to the diagnostic delay. Because the emergence of symptoms may precede fundoscopic manifestations, it is not uncommon to suspect functional visual loss with undetectable organic alteration^(17)^, as occurs in amblyopia.

The ERG was an objective and well-established resource to evaluate the visual function during the follow-up period of these patients^(27)^. During the evaluation period, CHC-UFPR was an important referral center in this category of dystrophies. However, from the initial suspicion by the assistant physician to the proper referral to a specialist, there may have been a delay that contributed to the late diagnosis, especially in patients who came from more distant locations. In addition, although it is the most common type of juvenile-onset macular dystrophy, this pathology is rare in the practice of most ophthalmologists, which makes establishing the diagnosis difficult.

In this study, the difference between the mean age at the onset of symptoms and the mean age at diagnosis in a reference center was 7.3 years; other literature studies have reported a similar range^(28,29)^. This delay in diagnosis leads to an overload in the health system owing to more consultations and investigative procedures, which is especially detrimental in a limited-resources country.

The alteration of at least one of the ERG phases in 100% of the patients in this study conflicted with the data in the literature^(5,16)^ and can be explained by the fact that, at the time of the examinations, full-field ERG was one of the few tests available for diagnosis in our country. Thus, patients in the initial stages may not have been diagnosed early by other methods and were then diagnosed by the full-field ERG. The patients included in this study showed large retinal areas of macular atrophy that were a particular feature of this sample, which may explain altered ERG^(19)^.

Another relevant fact was that the alteration of both photopic and scotopic stages in the majority of patients showed that most of this sample had cones and rods dysfunction. This may be another sign of advanced disea se, as the first abnormalities to be detected in Stargardt disease are cone-selective (e.g., decreased amplitude of the photopic b-wave). On the other hand, deep and generalized alterations of the photopic and scotopic phases are present in the late course of the disease^(30)^.

As an example of the variation in ERG results according to the evolutionary stages of the disease, authors^(16)^ performed ERG in 162 patients categorized following the Fishman classification and demonstrated that in Stage 1, 26% of the patients presented subnormal amplitude in the simple flash photopic step and 32% presented subnormal response to the flicker; for the scotopic phase, 19% of the patients presented subnormal response at the rod response and the maximum response steps. In Stages 2 and 3, there was a higher prevalence of b-waves with subnormal amplitudes in the photopic and scotopic phases, and all patients in stage 4 presented alterations in the ERG results.

There are currently tests capable of allowing earlier detection of intraretinal changes, such as optical coherence tomography (OCT) and multifocal ERG^(24)^, as full-field ERG is usually normal at the onset of disease manifestations. However, full-field ERG provides relevant clinical information regarding the severity of the disease and maintains an important prognostic value, because patients with higher central scotoma progression rates had significantly worse scotopic b-wave amplitudes^(27)^.

A limitation of this study is that fluorescein angiography was not performed; as previously described, this exam is altered in the great majority of the patients who have the disease and therefore would improve the diagnostic capacity. Another limitation is that many patients were referred from other services for just performance of the ERG, making it impossible to analyze all the medical records of the patients with suspected Stargardt disease who were assessed at the hospital. The retrospective nature of the study also made it impossible for patients to be classified in stages according to the alterations present in the fundoscopy.

There is no current treatment available for Stargardt disease, although stem cells, gene therapy, and other interventions have been studied. Thus, the delineation of the clinical profile of these patients is important as a first step, so that new prospective studies taking into account new technologies and genetic mutation analysis can be performed. Unfortunately, none of the aforementioned therapies has yet been able to safely and effectively treat a considerable number of patients^(9)^.

In the patients in this study, the majority presented within the first or second decade of life, VA was quite variable, and the main findings at fundoscopy were beaten-bronze reflex, posterior pole spots associated with macular alteration, and RPE atrophy. The typical fundus appearance of Stargardt disease and altered ERG were more frequent because of the delayed diagnosis.

The authors concluded that the findings of this study corroborate the literature, and that it is important to combine clinical approaches and different modality tests to enhance diagnostic capacity.
